# Sex Differences in the Relationship between Excessive Alcohol Consumption and Metabolic Abnormalities: A Community-Based Study in Taiwan

**DOI:** 10.3390/nu14142957

**Published:** 2022-07-19

**Authors:** Liang-Jen Wang, Chih-Lang Lin, Yi-Chih Chen, Chemin Lin, Yu-Chiau Shyu, Chih-Ken Chen

**Affiliations:** 1 Department of Child and Adolescent Psychiatry, Kaohsiung Chang Gung Memorial Hospital, Chang Gung University College of Medicine, Kaohsiung 833, Taiwan; wangliangjen@gmail.com; 2 Liver Research Unit, Chang Gung Memorial Hospital, Keelung branch, Keelung 204, Taiwan; lion@cgmh.org.tw; 3 College of Medicine, Chang Gung University, Taoyuan 333, Taiwan; w210060@cgmh.org.tw (Y.-C.C.); 8902008@cgmh.org.tw (C.L.); 4 Department of Psychiatry, Chang Gung Memorial Hospital, Keelung 204, Taiwan; 5 Community Medicine Research Center, Chang Gung Memorial Hospital, Keelung 204, Taiwan; yuchiaushyu@gmail.com; 6 Department of Nursing, Chang Gung University of Science and Technology, Taoyuan 333, Taiwan

**Keywords:** substance use disorder, metabolic syndrome, survey, sex, obesity, psychiatry

## Abstract

Excessive alcohol consumption, as part of an unhealthy lifestyle, can contribute to metabolic abnormalities. This study investigated the sex differences in the relationship between excessive drinking and the risk of metabolic abnormalities. This community-based study included 3387 participants (age range: 30–103 years, mean age ± SD: 57 ± 13.5 years, 38.2% males) from the northeastern region of Taiwan. All participants completed a demographic survey and were subjected to blood tests. The risks of excessive drinking were evaluated using the Alcohol Use Disorder Identification Test (AUDIT). The results showed that males were at higher risks of obesity, hypertension, and hypertriglyceridemia, but at a lower risk of abdominal obesity than females. Males with hazardous drinking were at greater risks of hypertension, hyperglycemia, low serum levels of high-density lipoprotein cholesterol, and hypertriglyceridemia compared to those with no drinking. Females with hazardous drinking were at a greater risk of hypertension than those with no drinking. There was no interaction effect of sex and excessive drinking on the risks of metabolic abnormalities after controlling for demographics and lifestyle-related habits. Future studies are warranted to explore the sex-specific risk factors for metabolic abnormalities and to elucidate the mechanism underlying this association between alcohol consumption and metabolic abnormalities.

## 1. Introduction

Metabolic syndrome (MetS) refers to a collection of symptoms that increases the risk of insulin resistance along with abnormal adipose deposition and function [1,2]. The diagnostic criteria for MetS include at least three of the following five medical conditions: high blood pressure, abdominal obesity, abnormally high fasting plasma glucose, elevated levels of serum triglycerides, and decreased levels of high-density cholesterol (HDL) [3]. Globally, MetS has become a serious public health problem, with an estimated prevalence of 20–25% in adults. MetS and its components are closely associated with increased risks of type 2 diabetes mellitus, cardiovascular disease, stroke, and mortality [4]. Numerous lifestyle-associated risk factors, such as excessive alcohol consumption, are linked to MetS [5]. High alcohol consumption results in a higher risk of developing hypertension, central obesity, and insulin resistance, the key elements of MetS [6,7].

Excessive alcohol consumption is toxic to almost every tissue of the body [8]. Previous meta-analyses revealed that alcohol consumption affects MetS in a dose-dependent manner in that high alcohol consumption increases the risk of MetS. In contrast, very light alcohol consumption may be linked to a reduced risk of MetS [9,10]. Another meta-analysis demonstrated that approximately 20% of individuals with alcohol use disorder have MetS [11]. However, the association between alcohol consumption and MetS can vary across countries or ethnicities. For example, alcohol consumption significantly affected the risk of MetS among males in China [12], Japan [13,14], and Korea [15]. However, after adjusting for sociodemographic factors, no significant association was found between drinking alcohol and MetS risk among African-Americans [16]. Among Caucasian males, all levels of alcohol consumption exhibit significant inverse associations with the incidence of MetS. This association is particularly observed among overweight and/or obese individuals [17].

Moreover, evidence suggests that the predictors of MetS are sex-specific. For example, females may be more vulnerable to developing MetS under work stress and with low socio-economic status [18]. A previous study in Taiwan demonstrated that lower muscle mass is associated with a higher risk of MetS among older individuals, particularly in females [19]. In Malta, males are at a higher risk of MetS; however, the association between alcohol consumption and MetS development is only significant among females [20]. Another study conducted in Australia revealed that daily drinking was associated with reduced cardiovascular and coronary heart disease mortality among females but not males [21]. Taken together, sex-related factors are sensitive to genetic background, physiological changes occurring around menopause, dietary habits, and psychosocial and cultural factors [18].

Compared to females, males are more prevalent to engage in problem drinking and develop alcohol use disorders [22]. However, excessive drinking among females may result in more medical problems. Sex-related biological factors, including alcohol pharmacokinetics and the levels of sex hormones, may contribute to such sex differences [23]. Sex differences may play a critical role in modulating the impacts of alcohol use on neurobiology, endocrine and cardiovascular systems [24,25]. Attention to sex specificity should be an indispensable prerequisite of clinical or epidemiological research on alcohol consumption and metabolic abnormalities to improve our understanding of this association and develop effective health strategies [26]. However, little is known about the effect of sex difference on the association between alcohol consumption and metabolic abnormalities.

This study investigated the relationship between alcohol consumption and metabolic abnormalities among the general population in Taiwan to fill the research gap. Furthermore, we explore whether sex is a modulating factor in such a relationship.

## 2. Methods

### 2.1. Participants and Procedures

The Institutional Review Board of the Chang Gung Memorial Hospital approved this research (IRB No: 103-3886C). All participants agreed to the study conditions and provided their informed consent before enrolling in this study.

In total, 3387 participants (age range: 30–103 years, mean age ± SD: 57 ± 13.5 years, 38.2% males) were enrolled. Participant recruitment and sample preservation were conducted at the Northeastern Taiwan Community Medicine Research Cohort (ClinicalTrials.gov Identifier: NCT04839796). The inclusion criteria were age >30 years and absence of pregnancy. All participants completed a questionnaire survey that assessed their demographics (including sex, age, education levels, marital status) and lifestyle-related habits (including smoking and drinking behavior). Further, physical examination included measuring the heart rate, blood pressure, body weight, body height, and waist girth (circumference). Body mass index (kg/m^2^) was calculated as weight (kg) divided by height (m) squared. Waist girth was measured at the midline between the lowest margin of the subcostal rib and the upper margin of the iliac crest.

### 2.2. Laboratory Analyses

The participants were also subjected to blood tests. Blood samples were obtained in the morning after overnight fasting, and the following parameters were measured in the central lab of the study site, as described in detail elsewhere [27,28]. Metabolic profiles were assessed through plasma levels of fasting glucose, triglycerides, total cholesterol, HDL-C and LDL-C. Blood samples were analyzed within 4 h after collection to determine complete biochemical parameters.

### 2.3. Assessment of Excessive Drinking

Risks of excessive drinking were evaluated using the Alcohol Use Disorder Identification Test (AUDIT) [29]. The AUDIT comprises a 10-item core questionnaire and an 8-item clinical procedure. The total score, ranging from 0 to 40, is based on the questionnaire alone and provides a reliable estimate of the severity of alcohol use behavior [30]. The international and Chinese versions of AUDIT were verified for their good psychometric properties [31,32]. In this study, risks of excessive drinking were categorized as no drinking (AUDIT score = 0), lower risk drinking (AUDIT score 1–7), and hazardous drinking (AUDIT score ≥ 8).

### 2.4. Assessment of Metabolic Abnormalities

A race-specific waist girth threshold, based on the NCEP ATP III criteria [33,34], was used to prevent distortions in MetS prevalence. Obesity was defined as BMI ≥ 27. The ATP III criteria defined MetS as the presence of at least three of the following five traits: abdominal obesity, determined on the basis of Asian waist circumference cut-offs (males: >90 cm, females: >80 cm); hypertension, defined as a blood pressure ≥130/85 mmHg or drug treatment for essential hypertension; a low HDL-C, defined as a serum high-density lipoprotein cholesterol (HDL-C) level <40 mg/dL (1 mmol/L) among males and <50 mg/dL (1.3 mmol/L) among females or drug treatment for low HDL-C; hypertriglyceridemia, defined as a serum triglycerides (TG) level ≥150 mg/dL (1.7 mmol/L) or drug treatment for elevated TG; and hyperglycemia, defined as a fasting plasma glucose level ≥100 mg/dL (5.6 mmol/L) or drug treatment for diabetes mellitus.

### 2.5. Statistical Analyses

Data were analyzed using the Statistical Package for the Social Sciences for Windows (version 16.0; SPSS, Inc., Chicago, IL, USA). Variables are presented as mean ± standard deviation or frequency. All statistical tests are two-tailed, and differences were considered significant at *p* < 0.05.

We used the Chi-square test (χ^2^) or Fisher’s exact test to compare the categorical variables between participants with different risks of excessive drinking. Furthermore, we used the one-way analysis of variance to compare the continuous variables between groups. Subsequently, multivariate logistic regression was used to determine the effects of excessive drinking on metabolic abnormalities while controlling for age, education levels, marital status and smoking. The metabolic profiles (obesity and each criterion of MetS) were set as dependent variables (categorical variables). The risks of excessive drinking and age of the participants were set as independent variables. Finally, we examined the interaction effects of sex and excessive drinking on metabolic profiles by logistic regression, controlling for age, education levels, marital status and smoking.

## 3. Results

This study included 3387 participants (mean age: 57 years, 38.2% male) from the northeastern region of Taiwan. Among all participants, 1863 (55%) did not drink, 1217 (35.9%) were low-risk drinkers, and 307 (9.1%) had hazardous drinking. Table 1 lists the comparison of alcohol consumption between males and females. Compared to females, male participants had a higher frequency of drinking and a higher frequency of hazardous drinking. In terms of types of alcoholic beverages, males more frequently consume beer, spirits, medicine liquor and other types of alcoholic beverages, whereas females more frequently consume cocktails. Compared to females, males had larger alcohol amounts consumed per occasion, defined by standard alcohol units (SAU).

Logistic regression, controlling for age, education levels, marital status and smoking revealed that males were at higher risks of obesity (aOR = 1.70, *p* < 0.001), hypertension (aOR = 1.65, *p* < 0.001), and hypertriglyceridemia (aOR = 1.49, *p* = 0.003), but at a lower risk of abdominal obesity (aOR = 0.71, *p* = 0.007) than females.

Table 2 summarizes the demographic characteristics, laboratory data, and metabolic profiles of the participants categorized by risks of excessive drinking. The non drinking group was older (*p* < 0.001). The hazardous drinking group had the highest proportions of males (*p* < 0.001), smoking (*p* < 0.001) and betel nut use (*p* < 0.001), and had the highest BMI (*p* < 0.001) and levels of AST (*p* < 0.001), ALT (*p* < 0.001), bilirubin (*p* = 0.016), r-GT (*p* < 0.001), AFP (*p* = 0.001), HbA1c (*p* = 0.002), uric acid (*p* < 0.001) and the lowest levels of adiponectin (*p* < 0.001) and leptin (*p* < 0.001). In terms of metabolic abnormalities, those with hazardous drinking had higher risks of hypertension (*p* = 0.003) and hyperglycemia (*p* = 0.001), hypertriglyceridemia (*p* < 0.001), and MetS (*p* = 0.004) compared to those with no drinking.

**Figure 1:**
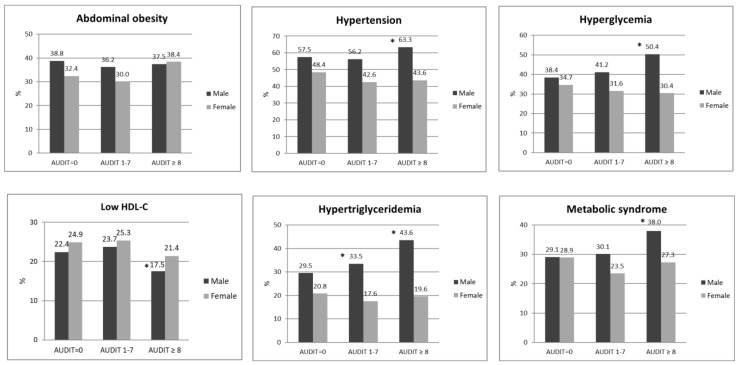
Proportion of each component of metabolic syndrome among males and females categorized by risks of excessive drinking. Note: The risks of excessive drinking were assessed using the Alcohol Use Disorder Identification Test (AUDIT): no drinking (AUDIT score = 0), low-risk drinking (AUDIT score 1–7), and hazardous drinking (AUDIT score ≥ 8). * *p* < 0.05 Significant difference of comparison to those with no drinking (AUDIT score = 0).

Figure 1 presents the proportion of each component of metabolic syndrome among males and females categorized by risks of excessive drinking. The proportion of males with abdominal obesity ranged from 36.2% to 38.8%, whereas that of females with abdominal obesity ranged from 30.0% to 38.4%. The proportion of males and females with hypertension ranged from 56.2% to 63.3% and 42.6% to 48.4%, respectively. The proportions of males and females with hyperglycemia ranged from 38.4% to 50.4% and 30.4% to 34.7%, respectively. The proportions of males with low HDL-C ranged from 17.5% to 23.7%, whereas the proportions of females with low HDL-C ranged from 21.4% to 25.3%. The proportions of males and females with hypertriglyceridemia ranged from 29.5% to 43.6% and 17.6% to 20.8%, respectively. The proportions of males and females with MetS ranged from 29.1% to 38% and 23.5% to 28.9%, respectively.

Table 3 lists the effects of excessive drinking on metabolic profiles by logistic regression, controlling for age, education levels, marital status and smoking. We found that males with hazardous drinking were at a greater risk of hypertension (aOR = 1.69, *p* = 0.003), hyperglycemia (aOR = 1.92, *p* < 0.001), low HDL-C (aOR = 0.63, *p* = 0.026), and hypertriglyceridemia (aOR = 1.45, *p* = 0.033) than those with no drinking. Males with low-risk drinking were at a greater risk of hyperglycemia (aOR = 1.43, *p* = 0.01) than those with no drinking. In females, those with low-risk drinking were at a greater risk of obesity (aOR = 1.25, *p* = 0.032) and hyperglycemia (aOR = 1.26, *p* = 0.042) than those with no drinking. Females with hazardous drinking were at a greater risk of hypertension (aOR = 2.07, *p* = 0.022) than those with no drinking. No interaction effect of sex and excessive drinking on the risks of metabolic abnormalities was observed after controlling for age, education levels, marital status and smoking.

## 4. Discussion

The large-scale sample in our study indicated that males were at higher risks of obesity, hypertension, and hypertriglyceridemia, but at a lower risk of abdominal obesity than females. Males with hazardous drinking were at greater risks of hypertension, hyperglycemia, low serum levels of high-density lipoprotein cholesterol, and hypertriglyceridemia compared to those with no drinking. Females with hazardous drinking were at a greater risk of hypertension than those with no drinking. There was no interaction effect of sex and excessive drinking on the risks of metabolic abnormalities after controlling for demographics and lifestyle-related habits.

It was found that frequent alcohol consumption contributed to a higher prevalence of MetS and its medical consequences in China [12] and Spain [35]. A Korean National Health Insurance Service-National Sample Cohort revealed that the major risk factors for MetS among individuals with physical disabilities were unhealthy lifestyle habits, obesity, and older age [36]. There was a significant dose–response relationship across beverage types, increasing apolipoprotein, adiponectin, and cholesterol levels [37]. The association between excessive drinking and increased risk of arterial hypertension was demonstrated worldwide [38]. Appetite regulation is essential in MetS neurobiology for the underlying mechanism. The dopaminergic reward system in the central nervous system also potentially plays a role in obesity and alcohol/drug addiction [39].

The association between hyperglycemia and alcohol consumption is controversial. Our study revealed that males with hazardous drinking were at increased risk of hyperglycemia. A Swedish study demonstrated outcomes comparable with ours [40]. High alcohol consumption increases the risk of abnormal glucose regulation in males. Compared with males, the associations are more complex in females: decreased risk with low or medium intake and increased risk with binge drinking [40]. However, a meta-analysis showed that moderate alcohol consumption decreases fasting insulin levels and HbA1c concentrations and increases insulin sensitivity [41]. The effect of alcohol consumption on MetS may be protective, detrimental, or J-shaped [8]. A previous study in Taiwan indicated that ethnicity plays a significant role in developing MetS. In particular, indigenous Tsou people had increased risks [42]. In sum, a complex mechanistic relation, such as ethnicity or sex, may play a role in the relationship between alcohol consumption and metabolic abnormalities.

Our results showed that males with hazardous drinking were at greater risks of hypertension, hyperglycemia, low se-rum levels of high-density lipoprotein cholesterol, and hypertriglyceridemia compared to those with no drinking. Females with hazardous drinking were at a greater risk of hypertension than those with no drinking. A study conducted in Malta reported that males are at higher risk of MetS, and MetS components are significant predictors along with alcohol habits [20]. A Korean survey demonstrated that alcohol drinking is the leading risk factor for hypertension, hypertriglyceridemia, and hyperglycemia in males. In females, the main risk factors are household income and educational level. The pattern of risk factors is sex-specific [43]. Another Korean study investigated the relationship between alcohol consumption and MetS in a large sample population, and the results showed that very light drinkers (<5 g/day) exhibit MetS prevalence for both males and females [44]. Our study showed that males with low-risk drinking were at a greater risk of hyperglycemia than those with no drinking and that low-risk drinking increased the risk of obesity in females. Smoking status and sex strongly influenced the association between long-term alcohol consumption and MetS and its components in terms of the amount of alcohol consumed [45,46]. Sex differences were also observed in the neural response to taste among people with obesity [47]. A number of studies are centered on the hypothesis that menopause-related hormonal changes ultimately affect the sex differences in prevalence and clinical expression of metabolic abnormalities [18]. A sex-specific interventional strategy should consider the risk factors for metabolic abnormalities and lifestyle management.

This study has several limitations. First, it is a cross-sectional study, and the causal relationship between metabolic abnormalities and alcohol consumption was not determined. A prospective study is warranted to clarify the relationships between metabolic abnormalities and alcohol consumption. Second, several important lifestyle factors, such as dietary habits, family history, physical activity and occupational could be associated with alcohol consumption and metabolic profiles [48,49] were not analyzed in this study. Further investigation is warranted to determine whether these factors have a confounding effect on the relationship. Third, alcohol consumption in this study was based on self-declaration and could be underreported by some participants. Therefore, the possibility of misclassification existed. Finally, our study did not reveal the benefits of low-risk drinking on metabolic profiles. Some studies presented benefits of moderate drinking on glucose, HDL and body weight [50,51]. The definition of low-risk drinking in this study is not the same as those of moderate drinking or light drinking in previous studies. The effects of light or moderate drinking on metabolic profiles need further investigations.

## 5. Conclusions

This community study based in Taiwan revealed that males were at higher risks of obesity, hypertension, and hypertriglyceridemia, but at a lower risk of abdominal obesity than females. Males with hazardous drinking were at greater risks of hypertension, hyperglycemia, low serum levels of high-density lipoprotein cholesterol, and hypertriglyceridemia compared to those with no drinking. Females with hazardous drinking were at a greater risk of hypertension than those with no drinking. There was no interaction effect of sex and excessive drinking on the risks of metabolic abnormalities after controlling for demographics and lifestyle-related habits. Future studies are warranted to explore the sex-specific risk factors for metabolic abnormalities and elucidate the mechanism underlying the association between alcohol consumption and metabolic abnormalities.

## Figures and Tables

**Table 1:** Comparison of alcohol consumption between males and females.

	Male (N = 1294)	Female (N = 2093)	*X* ^2^	*p*-Value
**Frequency of drinking**				
Once per month or less	825 (63.8%)	1868 (89.2%)	343.00	<0.001
2–4 times per month	166 (12.8%)	122 (5.8%)		
2–3 times per week	141 (10.9%)	60 (2.9%)		
4 times per week or more	162 (12.5%)	43 (2.1%)		
**Types of alcoholic beverages**				
Beer	167 (12.9%)	124 (5.9%)	49.62	<0.001
Spirits	186 (14.4%)	85 (4.1%)	115.54	<0.001
Cocktail	37 (2.9%)	184 (8.8%)	46.13	<0.001
Medicinal liquor	53 (4.1%)	71 (3.4%)	1.12	0.289
Others	221 (17.1%)	161 (7.7%)	70.41	<0.001
**Alcohol amount consumed per occasion** (standard alcohol units)			238.67	<0.001
***0–2***	1100 (75.5%)	2152 (93.3%)		
***3–4***	191 (13.1%)	92 (4%)		
***5–6***	82 (5.6%)	29 (1.3%)		
***7–9***	39 (2.7%)	14 (0.6%)		
***≥10***	42 (2.9%)	20 (0.9%)		
**Risks of excessive drinking**				
No drinking	478 (36.9%)	1385 (66.2%)	405.75	<0.001
Low-risk drinking	565 (43.7%)	652 (31.2%)		
Hazardous drinking	251 (19.4%)	56 (2.7%)		

**Table 2:** Comparison of demographics, lifestyle habits and metabolic profiles of 3387 participants categorized by risks of excessive drinking.

	No Drinking N = 1863 (55%)	Low-Risk Drinking N = 1217 (35.9%)	Hazardous Drinking N = 307 (9.1%)	*p*-Value
**Demographics and lifestyle-related habits**				
Male (%)	478 (25.7%)	565 (46.4%)	251 (81.8%)	<0.001
Age, years, mean ± SD	59.3 ± 13.5	54 ± 13.2	54.6 ± 11.7	<0.001
Education levels (%)				<0.001
Junior high school or lower	999 (54.3%)	466 (38.9%)	124 (41.1%)	
Senior high school	456 (24.8%)	350 (29.2%)	118 (39.1%)	
College or above	386 (21.0%)	382 (31.9%)	60 (19.9%)	
Marital status (%)				<0.001
Never married	114 (6.3%)	123 (10.4%)	18 (6.0%)	
Married	1446(79.5%)	944 (79.7%)	255 (84.7%)	
Divorced/Separated/Widowed	260 (14.3%)	117 (9.9%)	28 (9.3%)	
Smoke (%)				
Never	1554 (83.4%)	799 (65.8%)	76 (24.8%)	<0.001
Current	164 (8.8%)	217 (17.9%)	150 (48.9%)	
Previously	145 (7.8%)	199 (16.4%)	81 (26.4%)	
**Metabolic profiles (%)**				
Obesity (BMI ≥ 27)	766 (41.1%)	553 (45.5%)	159 (51.8%)	0.001
Abdominal obesity	691 (37.2%)	405 (33.3%)	117 (38.2%)	0.062
Hypertension	944 (50.8%)	595 (48.9%)	183 (59.8%)	0.003
Hyperglycemia	662 (35.7%)	439 (36.1%)	143 (46.7%)	0.001
Low HDL-C	452 (24.3%)	299 (24.6%)	56 (18.2%)	0.053
Hypertriglyceridemia	428 (23%)	304 (25%)	120 (39.2%)	<0.001
Metabolic syndrome	538 (28.9%)	323 (26.6%)	110 (36.1%)	0.004

**Table 3:** Effects of excessive drinking on metabolic profiles by logistic regression, controlling for age, education levels, marital status, and smoking.

Dependent Variables	Independent Variables	Female		Male	
	Risks of Excessive Drinking	aOR (95% CI)	*p*-Value	aOR (95% CI)	*p*-Value
Obesity	No drinking	1		1	
	Low-risk drinking	1.25 (1.02–1.54)	0.032 *	0.98 (0.76–1.26)	0.868
	Hazardous drinking	1.25 (0.70–2.26)	0.452	0.96 (0.69–1.33)	0.794
Abdominal obesity	No drinking	1		1	
	Low-risk drinking	1.19 (0.97–1.47)	0.100	0.88(0.67–1.16)	0.361
	Hazardous drinking	1.21 (0.66–2.22)	0.546	1.20 (0.85–1.69)	0.296
Hypertension	No drinking	1		1	
	Low-risk drinking	1.19 (0.96–1.47)	0.106	1.14 (0.88–1.49)	0.316
	Hazardous drinking	2.07 (1.11–3.88)	0.022	1.69 (1.20–2.37)	0.003
Hyperglycemia	No drinking	1		1	
	Low-risk drinking	1.26 (1.01–1.57)	0.042	1.36 (1.03–1.78)	0.028
	Hazardous drinking	1.66 (0.87–3.15)	0.119	1.92 (1.36–2.69)	<0.000 *
Low HDL-C	No drinking	1		1	
	Low-risk drinking	1.02 (0.81–1.28)	0.869	0.93 (0.69–1.26)	0.653
	Hazardous drinking	0.71 (0.35–1.42)	0.327	0.63 (0.41–0.95)	0.026
Hypertriglyceridemia	No drinking	1		1	
	Low-risk drinking	0.95 (0.74–1.22)	0.695	0.99 (0.75–1.30)	0.943
	Hazardous drinking	0.91 (0.43–1.94)	0.806	1.45 (1.03–2.03)	0.033
Metabolic syndrome	No drinking	1		1	
	Low-risk drinking	1.00 (0.79–1.26)	0.975	0.98 (0.74–1.30)	0.886
	Hazardous drinking	1.38 (0.71–2.68)	0.347	1.31 (0.93–1.85)	0.124

* *p* < 0.05; aOR, adjusted odds ratio; 95% CI, 95% confidence interval.

## Data Availability

Due to ethical restrictions, the data presented in this study are available on request from the corresponding author.
